# High-fat diet impairs duodenal barrier function and elicits glia-dependent changes along the gut-brain axis that are required for anxiogenic and depressive-like behaviors

**DOI:** 10.1186/s12974-021-02164-5

**Published:** 2021-05-16

**Authors:** Luisa Seguella, Mirella Pesce, Riccardo Capuano, Fabrizio Casano, Marcella Pesce, Chiara Corpetti, Martina Vincenzi, Daniela Maftei, Roberta Lattanzi, Alessandro Del Re, Giovanni Sarnelli, Brian D. Gulbransen, Giuseppe Esposito

**Affiliations:** 1 Department of Physiology and Pharmacology “V. Erspamer”, Sapienza University of Rome, Piazzale Aldo Moro 5, 00185 Rome, Italy; 2 Dept. of Physiology, Michigan State University, East Lansing, MI 48824 USA; 3 Department of Clinical Medicine and Surgery, University of Naples “Federico II”, 80131 Naples, Italy

**Keywords:** High-fat diet, Intestinal hyper-permeability, Enteric glia, Glial signaling, Gut-brain axis, Behavioral disorders

## Abstract

**Background:**

Mood and metabolic disorders are interrelated and may share common pathological processes. Autonomic neurons link the brain with the gastrointestinal tract and constitute a likely pathway for peripheral metabolic challenges to affect behaviors controlled by the brain. The activities of neurons along these pathways are regulated by glia, which exhibit phenotypic shifts in response to changes in their microenvironment. How glial changes might contribute to the behavioral effects of consuming a high-fat diet (HFD) is uncertain. Here, we tested the hypothesis that anxiogenic and depressive-like behaviors driven by consuming a HFD involve compromised duodenal barrier integrity and subsequent phenotypic changes to glia and neurons along the gut-brain axis.

**Methods:**

C57Bl/6 male mice were exposed to a standard diet or HFD for 20 weeks. Bodyweight was monitored weekly and correlated with mucosa histological damage and duodenal expression of tight junction proteins ZO-1 and occludin at 0, 6, and 20 weeks. The expression of GFAP, TLR-4, BDNF, and DCX were investigated in duodenal myenteric plexus, nodose ganglia, and dentate gyrus of the hippocampus at the same time points. Dendritic spine number was measured in cultured neurons isolated from duodenal myenteric plexuses and hippocampi at weeks 0, 6, and 20. Depressive and anxiety behaviors were also assessed by tail suspension, forced swimming, and open field tests.

**Results:**

HFD mice exhibited duodenal mucosa damage with marked infiltration of immune cells and decreased expression of ZO-1 and occludin that coincided with increasing body weight. Glial expression of GFAP and TLR4 increased in parallel in the duodenal myenteric plexuses, nodose ganglia, and hippocampus in a time-dependent manner. Glial changes were associated with a progressive decrease in BDNF, and DCX expression, fewer neuronal dendritic spines, and anxiogenic/depressive symptoms in HFD-treated mice. Fluorocitrate (FC), a glial metabolic poison, abolished these effects both in the enteric and central nervous systems and prevented behavioral alterations at week 20.

**Conclusions:**

HFD impairs duodenal barrier integrity and produces behavioral changes consistent with depressive and anxiety phenotypes. HFD-driven changes in both peripheral and central nervous systems are glial-dependent, suggesting a potential glial role in the alteration of the gut-brain signaling that occurs during metabolic disorders and psychiatric co-morbidity.

**Supplementary Information:**

The online version contains supplementary material available at 10.1186/s12974-021-02164-5.

## Background

Individuals with metabolic disorders such as obesity and type 2 diabetes (T2D) exhibit abnormal neuropsychological and behavioral functions, which may indicate shared pathological process [1]. Peripheral autonomic neural circuits monitor intestinal microbial and dietary contents and convey this information to the central nervous system (CNS) that, in turn, regulates behavior [2, 3]. The enteric nervous system (ENS) regulates moment-to-moment digestive functions and modulates metabolism through interactions with other autonomic neurons and central pathways [2]. Enteric neurons are altered by metabolic disorders and these changes include impaired inhibitory motor pathways in the duodenum that contributes to hyper-contractility and abnormal transmission between the gut and the brain [4]. The resulting effects lead to a failure in controlling glucose metabolism and contribute to insulin resistance [5].

Diabetics and individuals consuming a prolonged high-fat diet (HFD) develop low-grade inflammation in the small intestine that may compromise intestinal barrier function and alter the microenvironment within the ENS. Glial cell proliferation, nerve damage, and degeneration of inhibitory nitrergic neurons in the myenteric plexus have all been associated with intestinal dysmotility that occurs during the early stages of obesity [6]. Further prolongation of a HFD evokes depressive- and anxiety-like phenotypes associated with distinct alterations in the intestinal microbiome and brain metabolome that follow the disruption of intestinal barrier functions [7]. Based on this evidence, it is postulated that the increased translocation of microbes and their products across the impaired intestinal barrier negatively impacts the gut-brain circuits that control either energy metabolism or behaviors by establishing local neuroinflammation and enteric neuron dysfunction [6–8]. How neuropathological signaling triggered in the intestine reverberates through the nervous system is still unknown.

Homeostasis within the ENS is regulated by enteric glia through intercellular communication with neurons, immune cells, and the intestinal microbiota [3, 9]. Enteric glia constantly monitor their extracellular environment and exhibit physiological activity in response to extracellular signals such as neurotransmitters, microorganisms, and cytokines that function to modulate local intestinal functions and immune responses [10]. Proinflammatory insults and pathogens may also trigger a protective, reactive glial phenotype that has the potential to exacerbate local inflammatory responses and produce deleterious effects on neighboring cells [11, 12]. Enteric glial activation and proliferation are observed in animals consuming a HFD, and these glial changes are associated with intestinal motor disorders, an increased abundance of pro-inflammatory mediators [10], and a loss of inhibitory motor neurons within the myenteric plexus. Enteric glia also adopt a pro-inflammatory phenotype during neuroinvasion processes that occur when neurotoxic agents violate the intestinal epithelial barrier [13]. This appears to facilitate the ascension of neuronal dysfunction to the brain, thereby affecting neuronal survival, neurogenesis, and synaptic transmission [14, 15]. These observations suggest that glia may contribute to ENS dysfunction and changes to central processing in the context of metabolic disorders but the relative importance of glial changes remains untested.

The goal of this study was to test whether consuming a HFD drives a temporal progression of changes in glia and neurons along the gut-brain axis. Further, we hypothesized that enteric glia contribute to neuropathological signals that travel to the CNS and produce anxiogenic and depressive-like behaviors. We addressed these questions by blocking glial functions with the gliotoxin fluorocitrate (FC) and studied the effects of a prolonged HFD across the gut-brain axis that extends from the duodenal myenteric plexuses to the brain areas involved in the control of mood. Collectively, our data identified glial-mediated signaling triggered in the duodenal enteric plexuses as a critical link between peripheral and central neurocircuits that could be harnessed by treatments focused on correcting metabolic diseases and related neurobehavioral disorders.

## Methods

### Animals and experimental design

All experiments involving animals were carried out according to wit Sapienza University’s Ethics Committee. Animal care was in compliance with the IASP and European Community (EC L358/1 18/12/86) guidelines on the use and protection of animals in experimental research. Eight-week-old male C57BL/6 mice were used for the experiments (Charles River, Lecco, Italy). All mice were maintained on a 12-h light/dark cycle in a temperature-controlled environment with access to food and water ad libitum.

We used an accelerated and prolonged HFD protocol with 72% fat [6] to induce the onset of obesity/overweight after 4–6 weeks of diet ingestion [16]. Mice were fed with a standard chow diet containing 6.2% fat (SD group, *n* = 24; Charles River, Lecco, Italy), or with a HFD containing 72% fat (HFD group, *n* = 24; modified DIO 70% kcal fat diet with 2% additional corn oil, TestDiet, Richmond, IN) for 20 weeks (see scheme in Suppl. Figure 1A). To investigate whether enteric glia support HFD-induced peripheral and central nervous system impairment and neurobehavioral disorders development, we reversibly impaired enteric glial function using a protocol in which the gliotoxin fluorocitrate (FC) was administered daily through intraperitoneal injections. An additional group of mice fed with HFD was given daily intraperitoneal injections (IP) of FC (10 μmol/kg) for the entire duration of the study (HFD+FC group, *n* = 24). A group of control mice fed with a standard diet also received a daily IP with FC (10 μmol/kg) (SD+FC, *n* = 24) for 20 weeks to assess potential drug toxicity and the effects of impairing glial metabolism in healthy animals. Weekly body weight was measured to monitor the onset and progression of obesity. Behavioral tests were performed at weeks 0 and 20 of the diet protocol (days 0 and 140). All animals were sacrificed by cervical dislocation at 0, 6, and 20 weeks (*n* = 8 mice each time point), and the duodenum, nodose ganglia, and brain were removed to perform histological assessments. Immunofluorescence and immunohistochemistry analyses are described below. Neuronal cell cultures were obtained from duodenal myenteric plexuses and hippocampi collected from three mice for each experimental group at weeks 0, 6, and 20.

### Fluorocitrate solution preparation

FC solution was prepared as previously described [17]. Briefly, a clear solution of barium FC (Merck KGaA, Darmstadt, Germany) was obtained by dissolving the salt in half of the final volume of distilled water via sonication. Then, barium was separated from the solution as barium sulphate by adding a slight excess of sodium sulphate. This solution was filtered through a 0.2-μm filter, and distilled water was added to make up the final volume. NaCl was added to make the solution isotonic before use.

Enteric glial function was perturbed in vivo with a daily injection (IP) of FC (10 μmol/kg) for 20 weeks, according to a slightly modified reported method for enteric glia [18] and central glia. This concentration does not cause intestinal inflammation or neurodegeneration and no drug toxicity has been reported by prior studies that used this dose to impair glial function in the myenteric plexus [19].

### Body weight follow-up

Weekly body weight was recorded for each animal during the entire duration of the study. As healthy adult mice continued to grow throughout the study period, we compared the average weights of the HFD groups with the average weight of the SD group each week by 2-way ANOVA. HFD mice displayed statistically higher body weight at week 6 [16]; therefore, we considered 6 weeks as an intermediate time point to assess the impact of HFD on glia and neurons along the gut-brain axis. The gain in body weight as a proportion of initial body weight [initial weight (g)/total weight (g)] for each group was also assessed during the entire duration of the diet protocol to verify that weight changes were not influenced by initial animal weight.

### Histological analysis

The duodenum (the first 4–6 cm from the pyloric sphincter) was fixed overnight in ice-cold 4% paraformaldehyde (PFA, Merck KGaA, Darmstadt, Germany) and then transferred to a 1X PBS solution containing 20% sucrose at 4°C for 48 h. Duodenal segments were cut on a cryostat (15 μm) and stained with hematoxylin and eosin (H&E). Images were acquired through a ×10 objective (numerical aperture 0.25) with a high-resolution digital camera (Nikon Digital Sight DS-U1) using an inverted Nikon Eclipse 80i microscope (Nikon corporation, Minato, Tokyo, Japan) and analyzed offline using FIJI software (National Institutes of Health, Bethesda, MD).

Duodenal histopathological damage was scored at 0, 6, and 20 weeks by recording the individual score of (1) extent of villi damage, (2) mucosal damage degree, (3) inflammatory cell infiltration, (4) globet cell depletion, and (5) crypts absence according to the criteria previously described for histological intestinal alterations and summarized in the Table 1 [20]. The total histological damage score was expressed as average scores in each experimental group.

**Table 1:** Criteria definition in the evaluation of histomorphological scores for murine intestinal inflammation

Features	Scoring criteria	Score
Extent of mucosal villi damage	No damage	1
	Damage in <1/3 of villi	2
	Damage between 1/3 and 2/3 of villi	3
	Damage >2/3 of villi	4
Mucosal damage degree	No damage	1
	Swelling and loss of lamina propria matrix	2
	Loss of epithelium surface from villus tip to its base	3
	Loss of villi	4
Infiltration of Inflammatory cells degree	Normal	1
	Mild	2
	Moderate	3
	Dense	4
Goblet cells depletion	Absence	1
	Presence	2
Crypts absence	Absence	1
	Presence	2

### Circular muscle myenteric plexus (CMMP) whole-mount preparation

Segments of duodenum were immediately removed from euthanized mice at 0, 6, and 20 weeks and placed in ice-cold DMEM/Ham’s F-12 nutrient mixture (Corning, Kaiserslautern, Germany). The full-thickness tissues were then opened along the mesenteric border and pinned flat with mucosa facing-up in Sylgard-coated petri dishes (Dow Italia s.r.l., Milan, Italy). The mucosa was removed by cutting at the level of the lamina propria while separating the layers with forceps. Tissues were then flipped, and the longitudinal muscle and serosa were removed by microdissection to obtain live whole mounts with intact myenteric plexus lying atop of the preparation.

### Neuronal cells cultures

Neuronal cultures were obtained by microdissection from the duodenum or hippocampi of 3 mice at each time point according to slightly modified procedures previously described [21, 22] and briefly summarized below.

Duodenal segments were cleaned and pinned on plastic rods. The myenteric plexus and longitudinal muscle layers (LMMP) were separated from the underlying circular muscle by a cotton swab wetted with ice-cold Krebs moved along the entire line where the mesentery was attached. Thus, LMMPs were rinsed, snipped into tiny pieces by scissors, and then digested in carbogen-bubbled Krebs solution with 10X collagenase type 2 (Merck KGaA, Darmstadt, Germany), 10X protease (Merck KGaA, Darmstadt, Germany), and 5% bovine serum albumin (BSA, Serva, Heidelberg, Germany) for 30 min at 37°C [21]. The enzyme reaction was stopped by an ice-cold Krebs supplemented with 10% fetal bovine serum (FBS, Biowest, Nuaillé, France) and cell suspensions were collected following centrifugation at 500×*g* 8 min and plated onto poly-d-lysine- and laminin-coated coverslips in 6-well culture plates (Corning, Kaiserslautern, Germany). Neurobasal A medium (Life Technologies Italia, Monza, Italy) supplemented with 2% B-27 supplement (Life Technologies Italia, Monza, Italy), 1% heat-inactivated FBS, 1% Glutamax 100X (Life Technologies Italia, Monza, Italy), 10 ng/ml GDNF factor (Cell Guidance System Ltd, Cambridge, United Kingdom), and 1% antibiotic-antimycotic mixture (Merck KGaA, Darmstadt, Germany) was added to each well.

Mouse brains were placed into a dissection dish with Hibernate A (Life Technologies Italia, Monza, Italy) at 4 °C for hippocampus dissection [22]. Then, hippocampal slices (around 0.5-mm) were cut with a razor blade and transferred into a 30 °C pre-heated tube with 2 mg/ml papain (Merck KGaA, Darmstadt, Germany) in Hibernate A supplemented with 2% B-27 supplement and 0.5 mM Glutamax 100X (HABG). After 30 min, slices were triturated and fractions enriched in neurons were collected from the cell suspension. These were resuspended in pre-heated Neurobasal A supplemented with 2% B-27 supplement, 0.5 mM Glutamax 100X, growth factors (5 ng/ml mouse FGF2 and 5ng/ml mouse PDGFbb, Merck KGaA, Darmstadt, Germany), and 10 μg/ml gentamycin, Merck KGaA, Darmstadt, Germany) and plated onto poly-d-lysine- and laminin-coated coverslips in 6-well culture plates (Corning, Kaiserslautern, Germany). Subsequently, neurobasal A supplemented with 2% B-27 supplement, 0.5 mM Glutamax 100X, and appropriate growth factors were added to the cells. After 10 days, enteric and hippocampal neuronal cultures were fixed for 30 min in 4% PFA and labeled for PSD 95 to characterize dendritic spine density by immunofluorescence as described below [21, 22].

### Nodose ganglia dissection

As previously described [23], access to the nodose ganglia is provided by a midline longitudinal cut at the neck using surgical scissors. After sternohyoideus and omohyoideus muscles were separated, the internal carotid artery and vagus nerve were exposed. The vagus nerve enters the posterior lacerated foramen at the base of the skull and immediately displays a swelling of the nerve that is the nodose ganglion. Using the forceps, the vagus nerve was separated from the arteries and nodose ganglion was cleaned of any connective tissue. Thus, nodose ganglion were collected and fixed as described below.

### Immunofluorescence

Samples of duodenum and brain were fixed overnight in ice-cold 4% PFA, rinsed in 1X PBS for 30 min, and then transferred to a 1X PBS solution containing 20% sucrose at 4°C for 48 h. Tissue slices (15 μm) were cut on a cryostat and mounted onto poly-d-lysine-coated slides. After dissection, nodose ganglia were fixed for 30 min in ice-cold 4% PFA, rinsed in 1X PBS for 30 min, and then transferred to a 1X PBS solution containing 20% sucrose at 4°C for 48 h. Nodose ganglia were then mounted in OCT (optimal cutting temperature) compound, snap frozen in dry ice, and sectioned in 15-μm slices. All slices were collected onto SuperFrost Plus slides and air-dried at room temperature in the dark overnight before being immunolabeled. The duodenal CMMP preparations were fixed overnight in Zamboni’s solution at 4°C followed by a 30-min rinse in 1X PBS.

Tissues were rinsed three times (10 min each) in 1X PBS containing 0.1% Triton X-100 (T-PBS) followed by a 1-h incubation in blocking solution (containing 4% normal donkey serum, 0.1% Triton X-100, and 1% bovine serum albumin in 1X PBS) at room temperature. After three washes in T-PBS (10 min each), primary antibodies diluted in blocking solution were applied overnight at +4°C (Table 2). Tissues were rinsed three times (10 min each) with T-PBS after removing the primary antibodies, and then secondary antibodies were applied for 2 h at room temperature (Table 2). Tissues were rinsed two times (10 min each) in 1X PBS, once in 0.1 M phosphate buffer (10 min), and mounted in DAPI Fluoromount (SouthernBiotech, Birmingham, USA). Antibody specificity was confirmed by pre-adsorption with the corresponding control peptides. Images were acquired through the ×20 and ×40 oil immersion objectives (numerical aperture 0.50 and 0.75, respectively) using a high-resolution digital camera (Nikon Digital Sight DS-U1) on an inverted Nikon Eclipse 80i microscope (Nikon corporation, Minato, Tokyo, Japan) and analyzed offline using FIJI software (National Institutes of Health, Bethesda, MD) and Imaris 9.5.1 (Bitplane Inc) to segment and overlay pictures. Final images were transferred to Adobe Photoshop CS6 (Adobe Systems) for the construction of figure sets. Acquisition parameters were optimized to each tissue as the fluorescence intensity of each probe differed between tissues. After background subtraction, fluorescence intensity profiles for GFAP, TLR4, BDNF, HuCD, MAP-2, PSD 95, ZO-1, occludin, and DAPI were measured by FIJI software as maximum gray values within a manually defined region of interest in each image [arbitrary units (a.u.; mm^2^)]. Data were analyzed using GraphPad Prism 6 (GraphPad Software, San Diego, CA). Gray values were normalized per area unit in each image and expressed as relative fluorescence units (RFU) in comparison with the fluorescence intensities of the SD group at each time point. Neuronal dendritic spines’ number was expressed as average of the number of spines (PSD 95 immunoreactivity) along 10 μm of length versus the SD group.

**Table 2:** Primary and secondary antibodies used for immunofluorescence and immunohistochemistry labeling

Antigen	Host	Dilution	Source	Cat# / RRID
**ZO-1**	Rabbit	1:150	Bioss	bs-1329R
**Occludin**	Mouse	1:100	Invitrogen - Thermofisher scientific	33-1500
**CD 45**	Rabbit	1:500	Abcam	Ab10558
**GFAP**	Rabbit	1:500	ATLAS	HPA056030
**TLR4**	Mouse	1:250	Invitrogen - Thermofisher scientific	MA5-16216
**BDNF**	Rabbit	1:200	Bioss	BS-4989R
**HuCD**	Mouse	1:15	Invitrogen - Thermofisher scientific	A-21271
**MAP-2**	Rabbit	1:1000	Abcam	ab32454
**PSD 95**	Mouse	1:200	Invitrogen	7E3-1B8
**Anti-Rabbit IgG (H+L) [FITC]**	Donkey	1:1000	Novusbio	NB120-6798
**Biotin-SP (long spacer) AffiniPure F(ab')**_**2**_ **Fragment Anti-Rabbit IgG (H+L)**	Donkey	1:3000	Jackson ImmunoResearch	711-066-152
**Anti-Mouse IgG (H+L) [Texas Red]**	Goat	1:500	Novusbio	NB120-6787

### Immunohistochemistry

Fixed duodenal segments were cut into 15-μm-thick serial sections and mounted onto slides. After heat-mediated antigen retrieval, the tissue was blocked 1 h at room temperature with blocking solution (0.1% Triton X-100 and 2% Normal Donkey Serum in 0,1M Phosphate Buffer (PB) pH 7,4) and then incubated overnight with the primary antibody (Table 2). After three 10-min washes, the biotinylated secondary antibodies (Table 2) were added and samples were incubated 2 h at room temperature. Vectastain ABC Kit (Vector Laboratories, Burlingame, CA, USA) was added and samples were incubated at room temperature with Dab substrate working solution for 1 min (Vector Laboratories, Burlingame, CA, USA). The reaction was terminated in water and sections were then counterstained with hematoxylin-eosin. Negative controls were performed by omitting the primary antibody. Slides were analyzed with a microscope (Nikon Eclipse 80i by Nikon Instruments Europe) and images were captured at ×20 magnification by a high-resolution digital camera (Nikon Digital Sight DS-U1). Results were expressed as an average number of positive cells per mm^2^ assessed in 120/130 apical microvilli/ crypts.

### Behavioral tests

Behavioral tests were performed on days 0 and 140 of diet protocol and they were scheduled to avoid bias effects from the prior testing experience. Mice were sacrificed at the end of each set of experiments for further evaluations.

#### Open field test

An open field test was carried out to assess anxiety-like behavior. Each mouse was placed in the center of the open field arena of black Plexiglas (40 cm × 40 cm) and locomotor activity was recorded for 20 min by an overhead camera. After a 10-min acclimation, the remaining 10 min was used for assessment of anxiety-like behavior by recording the total mobility and immobility time (in seconds), and the number of entries in the center of the open field (20 cm × 20 cm delineate an area at 10 cm away from each wall). We considered an entrance into the center when the animal’s head and 80% of its body were within the delineated area.

#### Tail suspension test and forced swimming test

Depressive-like behavior was assessed by tail suspension and forced swimming tests. In the first test, mice were suspended by the tail on a horizontal bar (at around 40 cm from the floor) with adhesive tape, and immobility time (in seconds) was recorded during the last 4 min of the 6-min test. Mice were considered motionless when they did not exhibit escape-oriented behavior and passively hung without any body movements. In the forced swimming test, mice were placed in a cylinder (30 cm × 45 cm) filled with 27°C heated water, and immobility time (in seconds) was recorded during the last 4 min of the 6-min test. The immobility period was considered when animal floated in the water without behavior oriented to find an exit or made only the necessary movements to keep his head out of the water.

### Statistical analysis

Statistical analysis was performed by 2-way ANOVA when overall significant difference in gained weight and marker expression for treatment effect was found at each time point, with Dunnett’s multiple comparison post hoc test. One-way ANOVA with Dunnett’s multiple comparison post hoc test was used to assess difference in the histological damage score, the number of CD45 positive cells within the duodenal microvilli, and neuronal dendritic spines number among the different groups. Two-way ANOVA with Tukey’s multiple comparison post hoc test was used for behavioral studies comparing the treatment effect within the same group at distinct time points and among different groups at the same time point. Results are shown as means ± standard error of the mean (SEM) of 80 cells, 30 ganglia, and 8 mice for each experimental group at each time point; a *P* value < 0.05 was considered statistically significant.

## Results

### HFD impairs duodenal barrier function independent of glial activity

Metabolic disorders promote low-grade intestinal inflammation in the small intestine and alter intestinal permeability [5]. The ENS controls the intestinal epithelial activity and enteric glia may support barrier function through glial secreted factors [24, 25]. Given that a low-grade inflammatory state causes remodeling of the neuroepithelial compartment and promotes glial activity [26], we assessed duodenal barrier integrity along the 20 weeks of HFD protocol and disrupted glial function by FC (study protocol summarized in Additional file 1A).

**Fig. 1:**
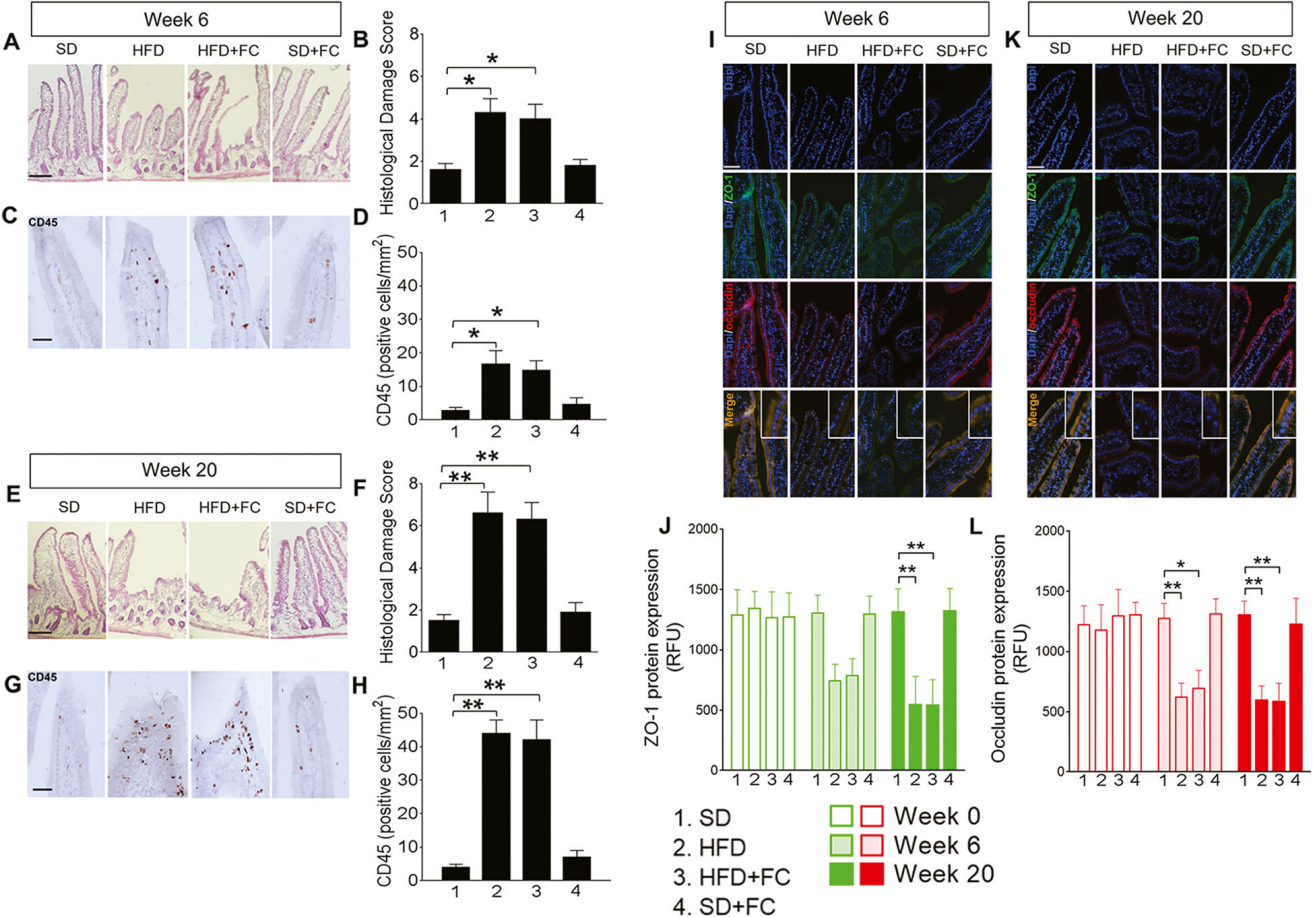
HFD evokes an intestinal inflammation and impairs duodenal mucosa without enteric glia involvement. Mice were fed with a standard diet (SD) or 72% high-fat diet (HFD) for 20 weeks, alone or with a daily IP of 10 μmol/kg fluorocitrate (FC) to investigate the enteric glia involvement in HFD-induced neuropathology. Representative images of duodenal cross-sections stained by hematoxylin and eosin with relative total histological damage score at weeks 6 (**a**, **b**) and 20 (**e**, **f**) of the diet protocol are shown, respectively. Representative images showing infiltrated CD45-positive cells within the duodenal microvilli and crypts and relative quantification at weeks 6 (**c**, **d**) and 20 (**g**, **h**). Immunoreactivity for Dapi (blue), ZO-1 (green), and occludin (red) and immunolabeling quantification for ZO-1 and occludin are also shown at weeks 6 (**i**, **j**, and **l**) and 20 (**k**, **j**, and **l**) of the diet protocol, respectively. Inserts within the merge pictures are enlarged areas. Data were analyzed by 2-way ANOVA or 1-way ANOVA and Dunnett post hoc. Results are expressed as cumulative histological damage, the average number of CD45+ cells or the average relative fluorescence units (RFU) ± SEM per area unit of *n* assessments. *P<0.05 and **P<0.01 versus relative SD group. Scale bars = 10 and 30 μm

Animals consuming a HFD exhibited increased body weight after 6 weeks of diet (*p*<0.1 at week 6 for HFD and HFD+FC *vs* SD group, 2-way ANOVA; Additional file 1B). Mice continued to gain weight throughout the study and body weight was not affected by FC treatment in control or HFD groups (*p*<0.05 at week 7, *p*<0.01 at weeks 8 and 9, *p*<0.001 at weeks 10–12, *p*<0.0001 at weeks 13–20 for HFD and HFD+FC groups *vs* SD group, 2-way ANOVA; Additional file 1B). Histological analysis showed epithelial barrier impairment in the duodenum of HFD mice at week 6 with a loss of mucosal integrity (*p*<0.05 *vs* SD group, 1-way ANOVA; Fig. 1a, b) and marked infiltration of inflammatory cells in the mucosa and microvilli in comparison to the SD group and baseline measurements at week 0 (*p*<0.05 *vs* SD group; 1-way ANOVA; Fig. 1c, d and Additional file 2). Histological damage scores and immune cell infiltration were greater in HFD mice at week 20 (*p*<0.01 *vs* SD group, 1-way ANOVA; Fig. 1e, f and g, h), and FC failed to counter such effects at both weeks 6 and 20 (*p*<0.05 at week 6 and *p*<0.01 at week 20 *vs* SD group, 1-way ANOVA; Fig. 1a–d and e–h). FC alone did not induce changes in epithelial architecture or affect the number of infiltrating immune cells in SD mice, confirming that no drug-related toxic effects occurred (Fig. 1a–d and e–h). Immunofluorescence analysis confirmed that animals consuming a HFD exhibited impaired duodenal barrier function as reflected by a severe loss in both zonula occludens (ZO-1) and occludin expression which was progressively observed at weeks 6 and 20 in comparison to SD group and week 0 (*p*<0.05 and *p*<0.01 for occludin at week 6, and *p*<0.01 for ZO-1 and occludin at week 20 *vs* SD group at the same time point, 2-way ANOVA; Fig. 1i–l, and Additional file 2). FC did not prevent the HFD-induced changes in epithelial barrier integrity and did not produce significant effects in the SD group at each time point (Fig. 1i–l).

### HFD elicited glial-mediated phenotypical changes in duodenal myenteric plexus and enteric neurons

Enteric glia monitor extracellular signals and enact responses that are aimed at preserving homeostasis [9, 27]. Enteric glia act as resident antigen-presenting cells and upregulate expression of major histocompatibility complex II (MHCII) and TLR4 in response to pathogenic microorganisms [28]. Enteric glia also release neurotrophins that support local immune responses [27]. However, reactive enteric gliosis involves the release of pro-inflammatory mediators that have potentially deleterious effects on neighboring cells [11, 29]. Given that HFD altered mucosa integrity in the duodenum, we hypothesized that changes in activated enteric glia could contribute to altered neuronal and immune responses.

**Fig. 2:**
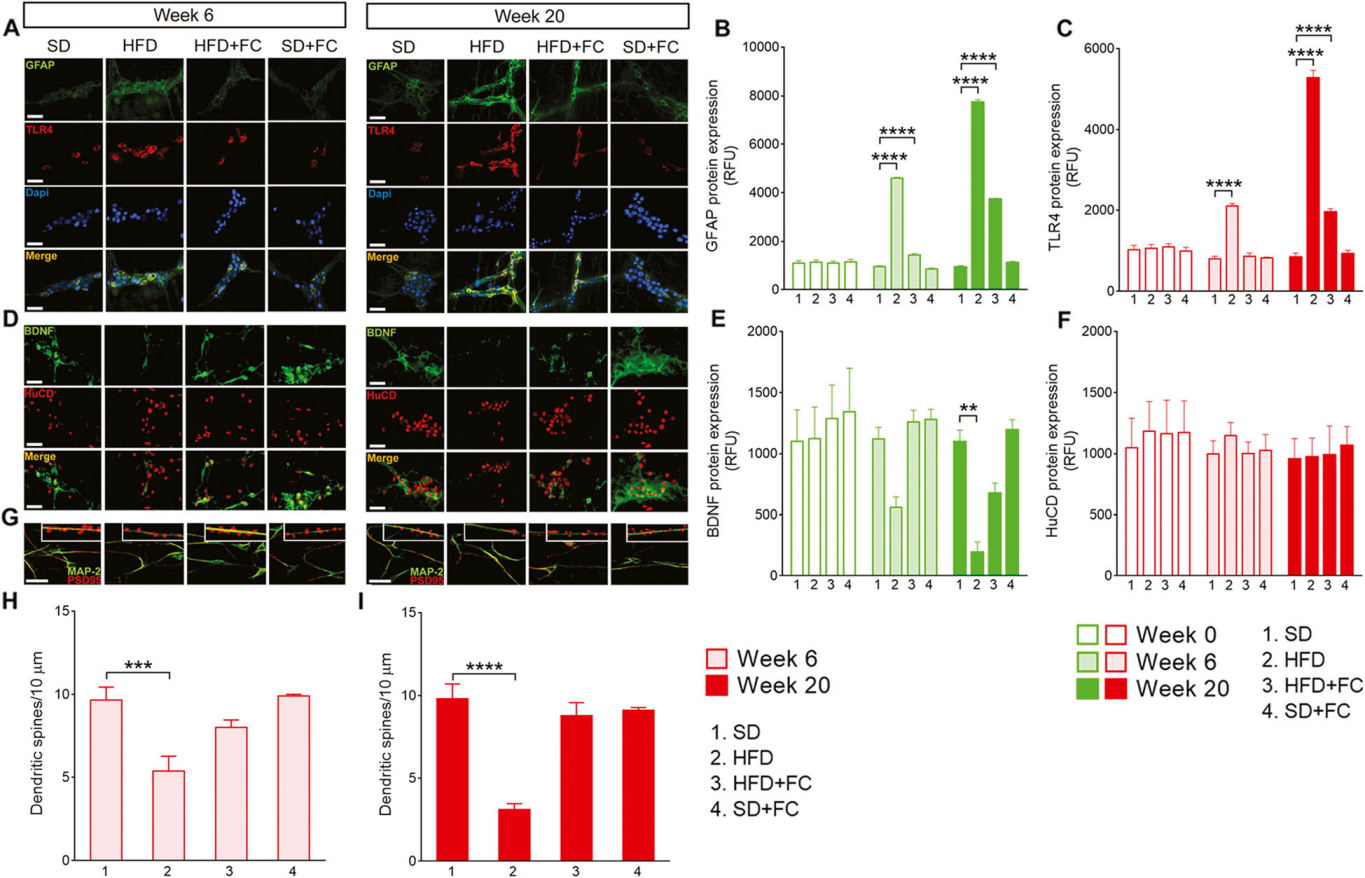
HFD induces glial-dependent changes in the duodenal myenteric plexus and dendritic spines decrease in neurons. Representative images of duodenal myenteric plexus from mice fed with a standard diet (SD) or high-fat diet (HFD) for 20 weeks, in the absence or presence of daily IP of 10 μmol/kg fluorocitrate (FC). GFAP (green), TLR4 (red), and Dapi (blue) immunoreactivity and relative immunolabeling quantification at weeks 6 and 20 (**a**–**c**) of diet protocol, respectively. BDNF (green) and HuCD (red) protein expression were also assessed at 6 and 20 weeks (**d**–**f**), respectively. The number of spines (PSD 95 immunoreactivity, red) was measured along neuronal dendrites (MAP-2 immunoreactivity, green) of cultured enteric neurons isolated from the duodenal myenteric plexuses of the above-mentioned experimental groups at weeks 6 and 20 (**g**–**i**) of diet protocol. Data were analyzed by 2-way ANOVA or 1-way ANOVA and Dunnett post hoc. Results are expressed as average relative fluorescence units (RFU) ± SEM per area unit or average number of dendritic spines/10 μm of *n* assessments. **P<0.01, ***P<0.001, and ****P<0.0001 versus relative SD group. Scale bars = 10 and 20 μm

HFD increased the expression of GFAP and TLR4 in the duodenal myenteric plexus by week 6 of the HFD (*p*<0.0001 for GFAP and TLR4 *vs* SD group, 2-way ANOVA; Fig. 2a–c, and Additional file 3). Changes in TLR4 expression were mitigated in FC-treated HFD mice (Fig. 2a–c). Glial expressions of GFAP and TLR4 were further elevated by week 20 (*p*<0.0001 for GFAP and TLR4 *vs* SD group, 2-way ANOVA; Fig. 2a–c) and FC did not prevent the increased GFAP and TLR4 upregulation (*p*<0.0001 for GFAP and TLR4 *vs* SD group, 2-way ANOVA; Fig. 2a–c). No significant changes in GFAP and TLR4 protein expression were observed in SD mice treated with FC at weeks 6 and 20 as compared to basal expression and the SD group (Fig. 2a–c and Additional file 3).

The ENS is gaining substantial interest as an early site of neuropathological changes that develop during extraintestinal pathologies in response to chronic stress or inflammatory stimuli [30]. Given that hippocampal BDNF expression decreases and enteric glia up-regulate TLR expression during inflammation [11, 28, 31–35], we tested whether the increased expression of TLR4 and GFAP within the duodenal plexuses correlated with changes in enteric neurons and BDNF expression in HFD mice. A time-dependent decrease in BDNF expression was observed in parallel with enteric glia activation in the HFD mice at weeks 6 (Fig. 2d and e) and 20 (*p*<0.01 *vs* SD group, 2-way ANOVA; Fig. 2d and e), without significant changes in the expression of HuCD (2-way ANOVA; Fig. 2d and f, and Additional file 3). FC countered significantly the reduction in BDNF expression in HFD animals at week 20 (2-way ANOVA; Fig. 2d and e). BDNF and HuCD expressions were not affected by FC treatment in SD mice, confirming that neurodegenerative effects did not occur at the selected FC dosage (Fig. 2d–f). Psychiatric disorders are associated with altered hippocampal expression of BDNF and proteins critical for dendritic spine maturation and formation such as PSD 95. Therefore, we tested the hypothesis that alterations in dendritic spines density also occur in enteric neurons during HFD, mirroring peripherally the scenario observed in the CNS during neurobehavioral diseases. Interestingly, enteric neurons collected from the duodenal myenteric plexus of HFD mice displayed a reduced number of PSD 95 spines on their dendrites (MAP-2) in culture after 6 (*p*<0.001 *vs* SD group, 1-way ANOVA; Fig. 2g and h) and 20 weeks (*p*<0.0001 *vs* SD group, 1-way ANOVA; Fig. 2g and i) on diet in comparison with neurons isolated at week 0 or from SD mice (Fig. 2g–i and Additional file 3).FC has protected against the reduction in dendritic spines in neurons collected from HFD mice without showing any effects in SD-derived neurons (Fig. 2g–i). Taken together, our data show that long-term HFD controls the development of features consistent with a reactive glial phenotype (increased GFAP and TLR4) and is responsible for the decrease in BDNF and synapse. These observations are in line with previous studies which showed that glia respond to microbes through TLR4 when barrier function is compromised and switch to reactive phenotype when the surrounding neuronal environment is altered [11, 28].

### HFD promotes glial-dependent changes in the nodose ganglia

**Fig. 3:**
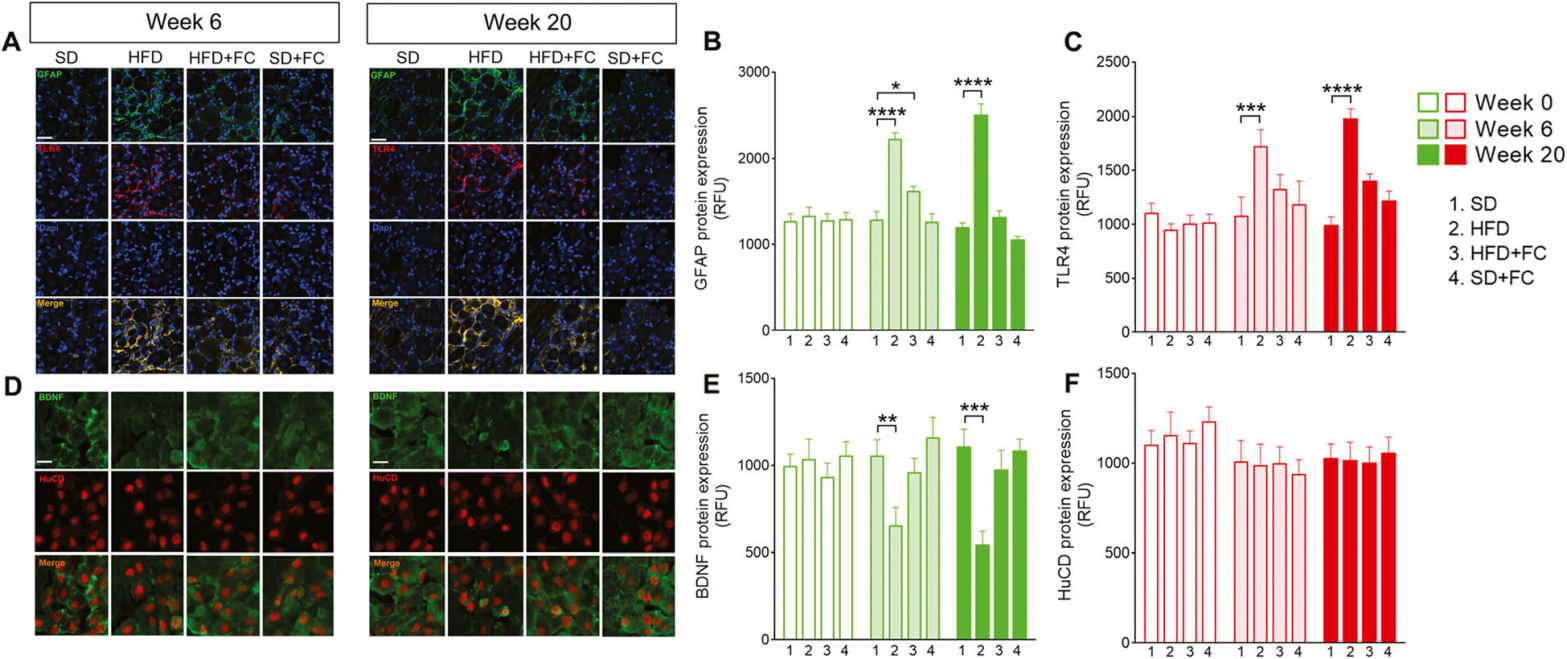
HFD evokes glial activation in the nodose ganglia after 20 weeks on diet. Immunofluorescence images show the expression of GFAP (green), TLR4 (red), and Dapi (blue) in the nodose ganglia and relative immunolabeling quantification at weeks 6 and 20 (**a**–**c**) of the diet protocol. BDNF (green) and HuCD (red) expression in nodose ganglia and relative quantification is shown at the same time points (**d**–**f**). Data were analyzed by 2-way ANOVA and Dunnett post hoc. Results are expressed as average relative fluorescence units (RFU) ± SEM per area unit of *n* assessments. *P<0.05, **P<0.01, ***P<0.001, and ****P<0.0001 versus relative SD group. Scale bar = 20 μm

Enteric glia undergo dynamic, reactive changes in response to injury and inflammation that profoundly alter their activity and lead to increased proliferation and a pro-inflammatory phenotype [36]. Reactive enteric gliosis occurs during inflammatory conditions in the intestine [11, 29] and neuronal dysfunctions [12] that, in certain circumstances, are not limited to ENS but reverberate to the CNS through the gut-brain axis [14]. To test whether HFD-induced glial reactivity was localized to the intestinal level or reverberated to the CNS, we performed the same immunohistochemical assessments carried out in the ENS in the nodose ganglia at 0, 6, and 20 weeks (Fig. 3 and Additional file 4). Immunofluorescence analysis revealed a significant upregulation of GFAP in the nodose ganglia of HFD mice at week 6 in comparison with SD mice and basal levels at week 0 (*p*<0.0001 *vs* SD group, 2-way ANOVA; Fig. 3a and b, and Additional file 4), which was maintained until the week 20 (*p*<0.0001 *vs* SD group, 2-way ANOVA; Fig. 3a and b). TLR4 expression was also markedly increased at weeks 6 (*p*<0.001 *vs* SD group, 2-way ANOVA; Fig. 3a and c) and 20 in HFD mice (*p*<0.0001 *vs* SD group, 2-way ANOVA; Fig. 3a and c), mirroring the scenario observed in the duodenal myenteric plexuses at the same time point. Conversely to what happened in the ENS, HFD mice treated with a daily IP injection of FC (10 μmol/kg) did not exhibit an increase in GFAP and TLR4 at weeks 6 and 20, respectively (Fig. 3a–c). Control SD mice treated with FC also exhibited no changes in GFAP or TLR4, suggesting that FC was able to prevent glial activation without evoking drug-related toxicity. Furthermore, BDNF expression progressively decreased in nodose ganglia from HFD mice and was significantly reduced at week 6 (*p*<0.01, 2-way ANOVA; Fig. 3d and e) and week 20 (*p*<0.001 *vs* SD group, 2-way ANOVA; Fig. 3d and e and Additional file 4). No significant changes in HuCD expression were detected at weeks 6 or 20 (Fig. 3d and f). Interestingly, FC treatment protected against the reduction in BDNF levels in HFD mice (Fig. 3d and e). FC did not alter BDNF in control SD mice, confirming that the protective effects of FC are strictly dependent on selective targeting of glia. Together, these results suggest that interfering with reactive gliosis may prevent neuropathological changes triggered by HFD both in the gastrointestinal tract and nodose ganglia.

### HFD is associated with reactive gliosis and glial-dependent neuropathological changes in the hippocampus

Enteric glia display a reactive phenotype from the earliest stages of extra-intestinal diseases such as Parkinson’s diseases (PD) and obesity, which are associated with central neurodegenerative processes and behavioral disorders, respectively [10, 16]. Furthermore, glial-mediated neuroinflammation propagates from the submucosal plexus through the spinal cord to the frontal cortex after intracolonic administration of HIV-1 Tat protein and ultimately impairs cognitive performance [14]. Given that HFD is associated with brain inflammation and glial reactivity within 24 h [37], we hypothesized that HFD-mediated neuropathological changes observed in the ENS and nodose ganglia could reverberate to the brain, affecting the brain areas involved in the control of behavior and mood.

**Fig. 4:**
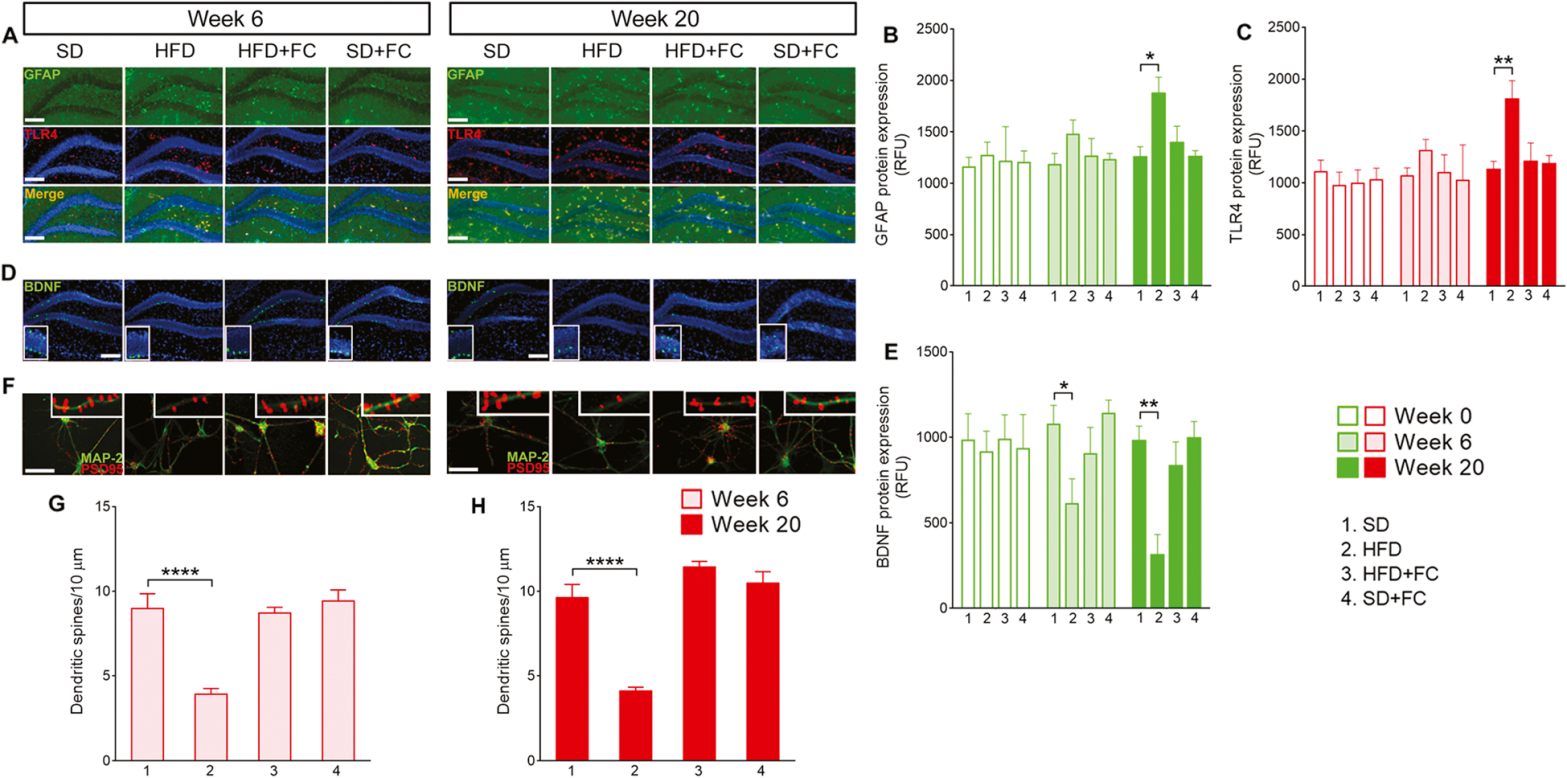
HFD drives glial-dependent changes in the hippocampus and neuronal dendritic spines decrease. Representative images of hippocampal slices from mice fed with a standard diet (SD) or high-fat diet (HFD) for 20 weeks, in the absence or presence of daily IP of 10 μmol/kg fluorocitrate (FC). Triple-label immunofluorescence for GFAP (green), TLR4 (red), and Dapi (blue) and related fluorescence intensity quantification at weeks 6 and 20 (**a**–**c**) of diet protocol are shown, respectively. In parallel, BDNF protein expression (green) in the dentate gyrus was assessed at 6 and 20 weeks (**d** and **e**), respectively. The number of dendritic spines (PSD 95 immunoreactivity, red) was measured in hippocampal neurons’ dendrites (MAP-2 immunoreactivity, green) isolated from the abovementioned experimental groups at weeks 6 (**f** and **g**) and 20 (**f** and **h**) of diet protocol. Data were analyzed by 2-way ANOVA and 1-way ANOVA and Dunnett post hoc. Results are expressed as average relative fluorescence units (RFU) ± SEM per area unit or the average number of dendritic spines/10 μm of *n* assessments. *P<0.05, **P<0.01, and ****P<0.0001 versus relative SD group. Scale bars = 10 and 20 μm

Similar to what we observed in the duodenal myenteric plexus and nodose ganglia, the expression of GFAP and TLR4 was significantly increased in the hippocampi of HFD mice at week 20 (*p*<0.05 for GFAP and *p*<0.01 for TLR4 *vs* SD group, 2-way ANOVA; Fig. 4a–c). FC treatment significantly inhibited this response, preserving GFAP and TLR4 expression similar to what was observed in SD mice and at week 0 (Fig. 4a–c and Additional file 5). SD mice that received FC treatment did not show any signs of reactive gliosis at the hippocampal level at each time point, confirming that FC did not cause the activation of central glia.

HFD evoked a progressive decrease in BDNF expression in the dentate gyrus of the hippocampus that paralleled changes in GFAP and TLR4 (*p*<0.05 at week 6 and *p*<0.01 at week 20 *vs* SD group, 2-way ANOVA; Fig. 4d and e; Additional file 5). HFD mice also exhibited reduced numbers of doublecortin X (DCX)-positive cells at week 20 (*p*<0.01 *vs* SD group, 1-way ANOVA; Additional file 6). Disrupting glial function with FC (10 μmol/kg) prevented the BDNF reduction and the loss of DCX-positive cells in HFD mice without causing significant effects in SD mice (Fig. 4d and e and Additional file 6). Furthermore, hippocampal neurons isolated from HFD mice at weeks 6 and 20 displayed a marked decrease in the number of dendritic spines (PSD 95 immunoreactivity) (*p*<0.0001 *vs* SD group at week 6 and *p*<0.0001 *vs* SD group at week 20, 1-way ANOVA; Fig. 4f–g; Additional file 5). FC preserved the number of dendritic spines in HFD-derived neurons at levels comparable to controls (Fig. 4f–h and Additional file 5) and FC itself had no effect on dendritic spine number in SD mice (Fig. 4f–h and Additional file 5).

Taken together, our results suggest that inhibiting the glial function in an intestinal inflammatory context during HFD prevents neuropathological alterations in the hippocampus.

### Behavioral disorders in HFD mice are glial-dependent

**Fig. 5:**
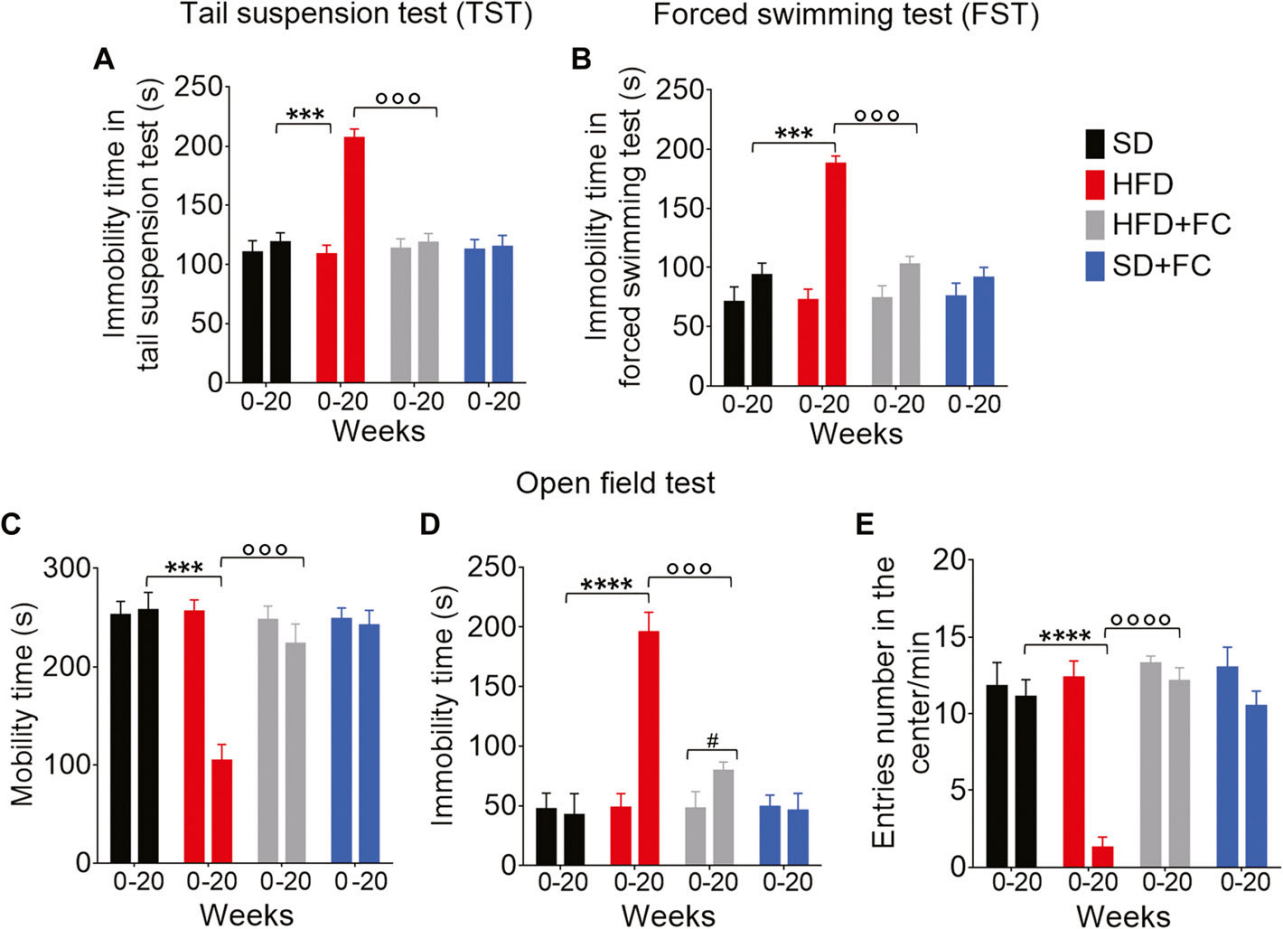
HFD mediates depressive- and anxiety-like behaviors by involving a glial signaling pathway. **a** Tail suspension and **b** forced swimming tests were performed at days 0 and 140 of the diet protocol to assess the depressive-like behavior. Mice were suspended by the tail on a horizontal bar or placed in a cylinder filled with heated water and immobility time (in seconds) was recorded during the last 4 min of the 6-min test. Open field test was carried out to assess anxiety-like behavior by recording the total **c** mobility and **d** immobility time (in seconds), and **e** the number of entries in the center of the open field during the last 10 min of the 20-min test. Data were analyzed by 2-way ANOVA and Tukey post hoc. Results are expressed as the average time in seconds or the average number of entries/min ± SEM of *n* assessments. ***P<0.001 and ****P<0.0001 versus SD group at day 140 (week 20), °°°*P*<0.001 and °°°°*P*<0.0001 versus HFD group at day 140 (week 20), #*P*<0.05 for day 0 versus day 140 within the same group

Metabolic disorders impair neuropsychological and behavior functions in obese and diabetic patients, suggesting common pathological processes [38]. In support, mice treated with long-term HFD exhibit depressive- and anxiety-like behaviors [39], although the mechanisms that negatively affect emotion are unresolved. Here, we tested whether HFD-evoked activation of glia along the gut-brain axis mediates the late development of behavioral dysfunctions. We performed tail suspension and forced swimming tests to investigate the development of depressive-like behaviors and open-field test to evaluate anxiety-like symptoms at days 0 and 140 (weeks 0 and 20). HFD mice exhibited increased immobility time in both tail suspension and forced swimming tests compared to SD mice at week 20 and their recorded time before starting the diet (*p*<0.001 *vs* SD group at week 20, 2-way ANOVA; Fig. 5a and b). Conversely, HFD mice treated with FC (10 μmol/kg) displayed immobility comparable with the SD group at week 20 and had no significant changes in their mobility from times recorded at day 0 (*p*<0.001 *vs* HFD group at week 20, 2-way ANOVA; Fig. 5a and b). FC had no significant effects on the immobility time of SD mice, confirming that behavioral alterations in HFD mice were mediated by glial responses rather than being related to the gliotoxin itself. Similarly, HFD mice spent significantly less time moving into an open field and consequently, increased their immobility compared to SD mice at week 20 and their recorded time at day 0 (*p*<0.001 and *p*<0.0001 *vs* SD group at week 20, 2-way ANOVA; Fig. 5c and d). This reduced the number of entries into the center during the recorded time (*p*<0.0001 *vs* SD group at week 20, 2-way ANOVA; Fig. 5e). FC treatment abolished the effects of HFD on behavior in the open field test at week 20 without causing significant effects in the behavior of SD mice (*p*<0.001 and *p*<0.0001 *vs* HFD group at week 20, 2-way ANOVA; Fig. 5c–e). The lack of depressive- and anxiety-like symptoms in HFD mice treated with FC indicates that behavioral alterations were mediated by glial activation along the gut-brain axis, rather than being related to the diet per se.

## Discussion

**Fig. 6:**
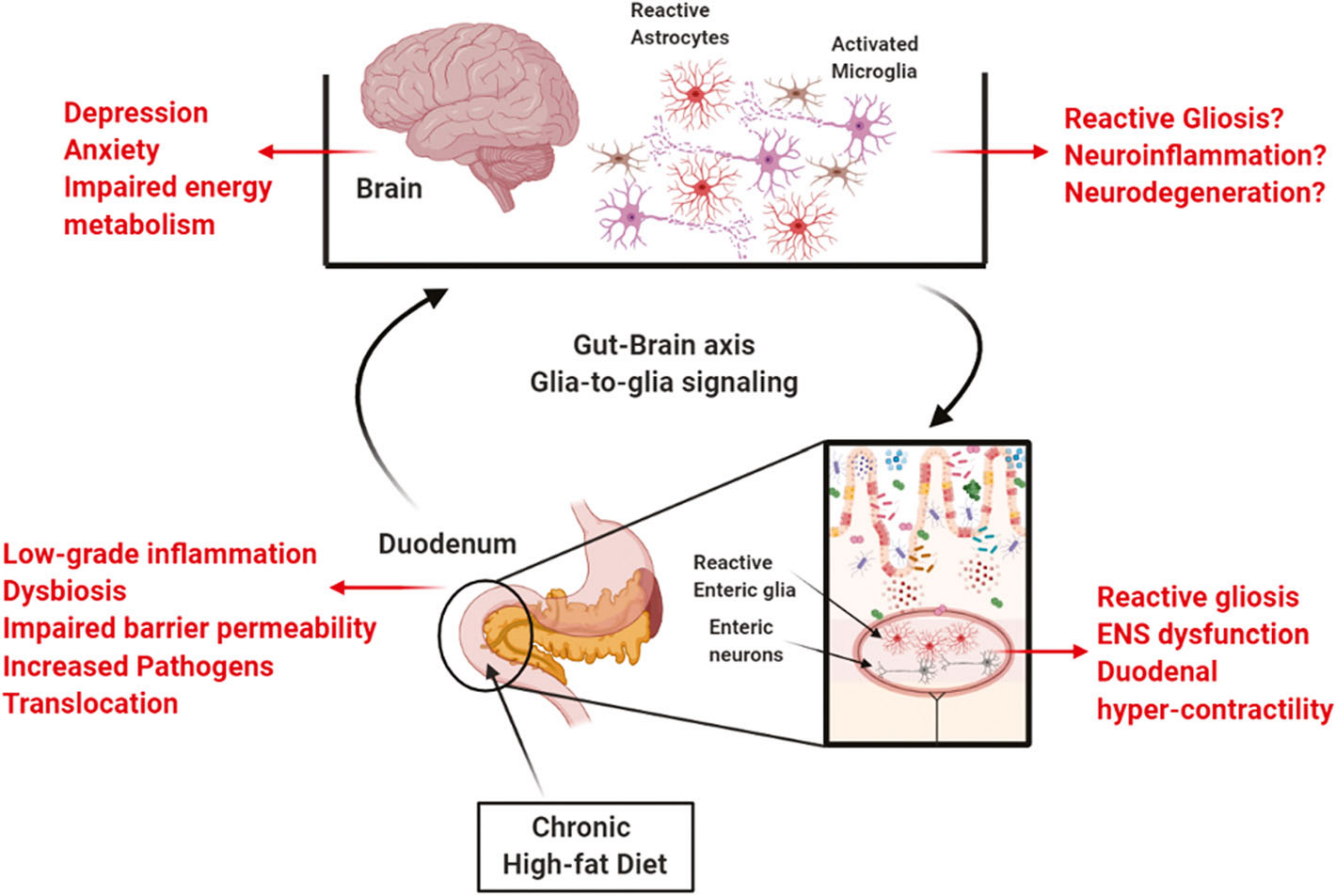
Schematic model of the gut-brain signaling during metabolic diseases and related behavioral disorders. Chronic exposure to high-fat diet (HFD) alters intestinal physiology, inducing a low-grade inflammation, dysbiosis, and increased mucosal permeability. Microbe-derived products that violate the impaired intestinal barrier may activate enteric glia within the enteric nervous system (ENS) via TLR4 pathway, leading to reactive gliosis and ENS neurons dysfunction. This elicits a glia-dependent signaling between the gastrointestinal tract and the brain responsible for impairing the energy metabolism and behavior, including depressive- and anxiety-like symptoms

Chronic low-grade inflammation in the proximal part of the intestine compromises duodenal barrier function, which “primes” an ENS dysfunction that appears to progress from the gut to the brain through nerve circuits that regulate energy metabolism and behavior [40]. Neurotoxic agents that violate the impaired intestinal barrier may elicit local neuroinflammation and affect ENS functions, including its ability to relay nervous messages to the brain [41]. Yet very little is known about how neuropathological signals are conveyed between the intestine and the brain and the effects of such neurotransmission during metabolic diseases. Here, we showed that a long-term HFD impairs duodenal barrier function and elicits glial-dependent signaling throughout the gut-brain axis that involves the GFAP/TLR4 network. This is paralleled by a decrease in BDNF expression that reverberates across the nervous system, a reduced number of dendritic spines in enteric and central neurons, and behavioral alterations. The effects in the nervous system and resulting behavioral changes were mitigated by perturbing glial function with the gliotoxin FC despite persistent intestinal barrier dysfunction. Taken together, our data suggest that glial-mediated signaling is crucial for conveying neuropathological signals across the nervous system and inducing psychiatric co-morbidity during metabolic disorders (Fig. 6).

Chronic exposure to HFD impairs glucose homeostasis and energy metabolism, inducing body weight gain, hyperglycemia, dyslipidemia, glucose tolerance decrease, and insulin resistance [16, 42]. HFD is also associated with modifications in the physiology of the small intestine with low-grade inflammation of the mucosa, decreased expression of tight junction proteins and high permeability, decreased antimicrobial peptides expression, impaired mucus production, and alterations to the functions of immune cells and ENS. Likewise, our results show that a progressive gain in bodyweight is associated with impaired duodenal mucosa integrity in HFD mice. This is reflected by decreased expression of tight junction proteins ZO-1 and occludin at 6 and 20 weeks (Fig. 1). Further, a time-dependent worsening of duodenal mucosa damage is associated with HFD. Notably, the degree of inflammation detected in our experimental condition did not reach the same magnitude as what is observed during inflammatory bowel diseases or severe infection, suggesting that HFD induced a low-grade inflammation at the duodenal mucosa level.

Neurons and glia communicate with each other to regulate ENS activities that control intestinal functions such as epithelial barrier function, secretions, and absorption [26, 43]. Enteric glial phenotype is profoundly altered in the context of inflammation or infection during which glia adopt a pro-inflammatory phenotype and display increased proliferation [29]. Reactive enteric glia may release pro-inflammatory factors, such as S100β, NO, IL-6, and IL-1β, which exacerbate the epithelial barrier dysfunction and damage [18, 36]. Thus, we tested the involvement of the glial component in mediating the duodenal mucosa damage that follows HFD exposure. Interestingly, interfering with glial functions induced by FC did not improve the epithelial expression of tight junction proteins and/or barrier damage in the duodenum of HFD mice (Fig. 1), suggesting that no glial-mediated mechanisms underlie such effects.

What role enteric glia serve in intestinal epithelial functions under physiological conditions is still an unresolved issue. Enteric glia regulate the expression of genes responsible for adhesion, differentiation, and proliferation of epithelial cells; promote mucosal healing by releasing proEGF and 11βPGF2 [24]; and protect the barrier integrity via S-nitrosoglutathione (GSNO) secretion both in physiological and pathological conditions [44]. Certain models of acute glial ablation profoundly affect barrier function and cause a substantial breakdown of the epithelium and an impaired release of glial factors which are also implicated with inflammatory bowel diseases [25, 44]. Conversely, depleting enteric glia in transgenic mice does not appear to change barrier function [43]. Moreover, S100β-mediate NO release by enteric glial cells is correlated with an increase in permeability and epithelial barrier dysfunction under different pro-inflammatory stimuli [18].

Such conflicting data likely reflect nuances in the specific experimental models of glial depletion and/or the pathophysiological state considered. It is also possible that enteric glia exhibit beneficial or detrimental effects on epithelial cells depending on the ability of glia to switch from a protective to a harmful role and vice-versa in response to specific stimuli. In our experiments, FC was engaged to alter glial activity in two distinct conditions: physiological (SD+FC group) and intestinal inflammatory (HFD+FC group) conditions in order to validate our experimental method and verify whether FC displayed distinct effects. The absence of effects in FC-treated SD mice strongly suggests that significant changes afterward observed in glia and neurons in HFD mice treated by FC were related to glial function.

The development of chronic inflammation leads to the subsequent loss of epithelium integrity, which fails to prevent the diffusion of microbes and other pathogens through the mucosa [5, 41]. Most microbe-derived products activate both epithelial cells and innate immune cells through pathogen recognition receptors such as toll-like receptors [45]. This may initiate an adaptive immune response and result in a massive release of pro-inflammatory cytokines that, in turn, modulate ENS activity either directly or through enteric glial cells [28]. The alterations of ENS functions contribute to impair gut-brain signaling leading to an imbalance of energy metabolism [5].

Enteric glia regulate bi-directional interactions between the immune and nervous systems by releasing neurotrophic factors that modulate innate immune responses [27], contribute to the activation of muscularis macrophage [46], modulate adaptive immune responses to pathogenic microorganisms by taking part in the process of immune recognition of detrimental stimuli via MHCII [28] and TLR-2 and 4 [11, 28], and convey the vagal anti-inflammatory signals to resident immune cells during injury and inflammation. Here, we showed that enteric glia in duodenal myenteric plexus express GFAP and TLR4 in response to increased duodenal permeability induced by HFD after 6 and 20 weeks of the diet (Fig. 2). This supports previous studies showing that enteric glia respond to pathogenic bacteria by upregulating immunomodulatory mechanisms via the TLR4 pathway and orchestrate a persistent enteroglial-sustained inflammation in the human intestine through S100β/TLR4 axis [11, 28].

In the context of inflammation, S100β overexpression by reactive glia induces the expression of different members of the TLR family in enteric glial cells, including TLR4 [28, 29]. TLR stimulation leads to glial transcriptional responses that involved shift in genes associated with the innate and adaptive immune responses [28], including overexpression of neurotrophins, growth factors, and cytokines. These mediators recruit infiltrating immune cells to the intestinal mucosa and support enteric glial-mediated neuroinflammation with deleterious effects on neighboring neurons [12, 29]. We observed a progressive reduction in BDNF expression in duodenal myenteric plexuses without changes in HuCD immunoreactivity. This BDNF decrease correlated with the increased glial expression of TLR4 and GFAP in the HFD mice after 6 and 20 weeks (Fig. 2). Although no significant changes in GFAP and TLR4 expression were observed in the duodenal myenteric plexues of FC-treated HFD mice, the ability of FC to counter BDNF decrease at week 20 suggests that enteric neuronal changes were, at least in part, glial-mediated.

In addition to modulating neuronal survival and synaptic functions, BDNF is an essential neurotrophin that regulates ENS signaling involved in the modulation of both sensory and motor functions. Alterations in the expression of BDNF in the gastrointestinal tract and brain are associated with impaired intestinal motility [47], psychiatric disorders, irritable bowel syndrome, and antibiotic-induced dysbiosis [33–35]. Interestingly, Bercik et al. [33] showed that enteric neurons are hyperexcitable during infectious colitis in mice and activate vagal pathways which signal to the CNS and contribute to low BDNF levels in the hippocampus and anxiety-like behaviors. In line with these results, we observed a progressive time-dependent decrease in BDNF expression in the duodenal myenteric plexus, nodose ganglia, and hippocampus during the 20 weeks of HFD (Figs. 3 and 4). This was associated with a progressive increase of GFAP and TLR4 expression across the nervous system. Conversely to what observed in the duodenum, FC prevented HFD-induced glial changes both in the nodose ganglia and hippocampus, suggesting that the effect of peripherally altered glial function reverberates along the gut-brain axis to a greater impact than the inflammation site. At the end of the diet protocol, HFD mice also exhibited reduced numbers of DCX-positive cells in the dentate gyrus of hippocampus concomitant with the development depressive- and anxiety-like behaviors at week 20 (Fig. 5 and Additional file 6). Furthermore, HFD induced a reduction in the number of dendritic spines, in terms of reduced expression of postsynaptic density protein 95 (PSD 95), both in cultured duodenal and hippocampal neurons at weeks 6 and 20 (Figs. 2 and 4).

Excitatory synapses encompass dense thickenings of proteins, referred to as postsynaptic densities (PSDs), consisting of glutamate receptors; cell adhesion proteins; scaffold proteins, such as PSD 95, PSD93, NF-L, and SAP102; and signaling molecules tightly associate via protein-protein interactions [48]. PSD 95 is known to be critical to the dendritic spine maturation and synaptic formation by controlling the ratio of excitatory to inhibitory synapses. In the hippocampus, an increased number of PSD 95-positive dendritic protrusions promotes excitatory synaptic differentiation, thereby resulting in regulating hippocampal synaptic transmission, plasticity, and hippocampus-dependent behavior [49]. Consistent with the hypothesis of altered excitatory glutamate neurotransmission, hippocampal abnormalities of glutamatergic receptors and NMDA receptor-interacting proteins, including PSD 95, were reported in several psychiatric disorders, such as schizophrenia, bipolar disorder, and depression [50].

Based on this background, we speculate that HFD-induced intestinal low-grade inflammation leads to duodenal barrier hyper-permeability and increases translocation of lumen pathogens able to potentially active glia in the myenteric plexus. The consequent neuronal dysfunction could result in increased excitability of enteric neurons and duodenal hyper-contractility. This would be responsible for sending abnormal nervous messages to the hypothalamus [4, 5], which fails to correctly control the energy homeostasis. The neuropathological signal conveyed between the gastrointestinal tract and the brain through a glial-mediated signaling alters the nervous circuits that control the behavior and mood at the hippocampal level, inducing the neurobehavioral disorders that are commonly observed in diabetic and obese patients (Fig. 6).

Although our study showed for the first time the role of enteric glia in mediating neuropathological signaling from the periphery to the brain and behavioral alterations during HFD-induced intestinal inflammation, there are some limitations that have to be considered. In our chronic model of HFD, we were not able to fully characterize biological changes occurring in activated enteric glia cells and astrocytes, respectively, and this would provide a potential therapeutic target for treating depressive disorders induced by metabolic diseases. The finding that hippocampus is involved in HFD-related behavioral alterations is in line with literature reports, but other brain regions have been involved in depressive disorders induced by metabolic diseases and we believe that should be necessarily investigated in the future.

Additional studies are thus required to clarify the role of glia in the context of mood disorders associated with metabolic diseases; our preliminary data suggest that glial component plays an important role in controlling the behavior and, thus, should be consider when we study the impact of gut-brain axis on energy metabolism and behaviors.

## Supplementary Information

**Additional file 1 **HFD increases body weight during 20 weeks of diet protocol. (A) Schematic representation of the experimental protocol with time schedule for immunohistochemistry assessments in the enteric and central nervous systems and behavioural tests. Mice were fed with a standard diet (SD) or 72% high-fat diet (HFD) for 20 weeks, alone or with a daily IP of 10 μmol/Kg fluorocitrate (FC) to investigate the enteric glia involvement in HFD-induced neuropathology. (B) Weekly body weight gain exhibited by standard diet (SD) and high-fat diet (HFD) mice during the 20 weeks of diet protocol. Data were analyzed by 2-way ANOVA and *Dunnettpost-hoc*. Results are expressed as average weights than their SD counterparts.

**Additional file 2 **Representative images of duodenal cross-sections stained before starting the diet protocol. Representative images of duodenal cross-sections stained by (A) hematoxylin and eosin or immunolabeled for (C) CD45 (brown) or (E) Dapi (blue), ZO-1 (green), and occludin (red) before starting the diet protocol. (B) Relative total histological damage score, (D) average number of CD45+ cells/mm^2^. Data were analyzed by 2-way ANOVA or 1-way ANOVA and *Dunnettpost-hoc*. Results are expressed as cumulative histological damage score ± SEM or the average relative fluorescence units (RFU) ± SEM per area unit of *n* assessments. Scale bars = 10 and 30 μm.

**Additional file 3 **Representative images of duodenal myenteric plexus and cultured enteric neurons before starting the diet protocol. In the duodenal myenteric plexus, (A) GFAP (green), TLR4 (red), and Dapi (blue) immunoreactivity were quantified together with (B) BDNF (green) and HuCD (red) expression at week 0 (relative immunolabeling quantification shown in Fig. 2). The number of spines (PSD 95 immunoreactivity, red) was measured along neuronal dendrites (MAP-2 immunoreactivity, green) of cultured enteric neurons isolated from the duodenal myenteric plexuses before starting the diet protocol (C and D). Data were analyzed by 2-way ANOVA or 1-way ANOVA and *Dunnettpost-hoc*. Results are expressed as average relative fluorescence units (RFU) ± SEM per area unit or average number of dendritic spines/10 μm of *n* assessments. Scale bars = 10 and 20 μm.

**Additional file 4 **Immunofluorescence images of nodose ganglia isolated from each group before starting the diet protocol. (A) GFAP (green), TLR4 (red), and Dapi (blue) in the nodose ganglia and relative immunolabeling quantification shown in Fig. 3. (B) BDNF (green) and HuCD (red) expression in nodose ganglia with relative quantification is also shown. Data were analyzed by 2-way ANOVA and *Dunnettpost-hoc*. Results are expressed as average relative fluorescence units (RFU) ± SEM per area unit of *n* assessments. Scale bar = 20 μm.

**Additional file 5 **Representative images of hippocampal dentate gyrus and cultured neurons before starting the diet protocol. (A) Triple-label immunofluorescence for GFAP (green), TLR4 (red), and Dapi (blue) with relative fluorescence intensity quantification shown in Fig. 4. (B) BDNF protein expression (green) and immunoquantification in the dentate gyrus of the hippocampus. (C and D) Representative pictures show the neuronal spines (PSD 95 immunoreactivity, red) measured along hippocampal neurons’ dendrites (MAP-2 immunoreactivity, green) isolated before starting the diet protocol. Data were analyzed by 2-way ANOVA or 1-way ANOVA and *Dunnettpost-hoc*. Results are expressed as average relative fluorescence units (RFU) ± SEM per area unit or the average number of dendritic spines/10 μm of *n* assessments. Scale bars = 10 and 20 μm.

**Additional file 6 **Immunofluorescence images showing the doublecortin X (DCX)-positive cells in the dentate gyrus of the hippocampus. Representative images for Dapi (blue) and DCX (green) with relative quantification of DCX-positive cells into the dentate gyrus area at (A-B) 0 and (C-D) 20 weeks. Data were analyzed by 1-way ANOVA and *Dunnettpost-hoc*. Results are expressed as average number of DCX-positive cells ± SEM in the dentate gyrus of *n* assessments. Scale bar = 100 μm.

## Data Availability

The datasets used and/or analyzed during the current study are available from the corresponding author on reasonable request.

## Abbreviations

ENS: Enteric nervous system
CNS: Central nervous system
SD: Standard diet
HFD: High-fat diet
ZO-1: Zonula occludens 1
FC: Fluorocitrate
T2D: Type 2 diabetes
GFAP: Glial fibrillary acidic protein
TLRs: Toll-like receptors
BDNF: Brain-derived neurotrophic factor
CMMP: Circular muscle myenteric plexus
LMMP: Myenteric plexus and longitudinal muscle layers
RFU: Relative fluorescence units
MHCII: Major histocompatibility complex
PSD 95: Postsynaptic density protein 95
GDNF: Glial cell-derived neurotrophic factor
DCX: Doublecortin X
