# Supplementary figures and images for: High-fat diet impairs duodenal barrier function and elicits glia-dependent changes along the gut-brain axis that are required for anxiogenic and depressive-like behaviors
Source: J Neuroinflammation. 2021 May 16;18:115. doi: 10.1186/s12974-021-02164-5 (PMC8126158; doi:10.1186/s12974-021-02164-5)

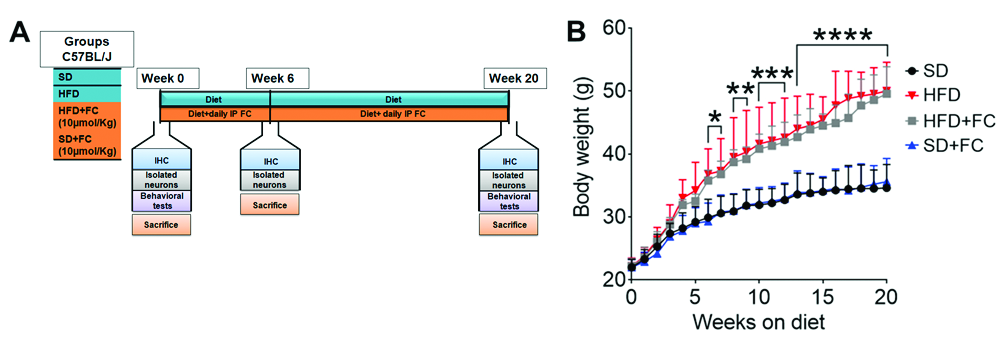

Supplement: Supplementary file 1 — Additional file 1 HFD increases body weight during 20 weeks of diet protocol. (A) Schematic representation of the experimental protocol with time schedule for immunohistochemistry assessments in the enteric and central nervous systems and behavioural tests. Mice were fed with a standard diet (SD) or 72% high-fat diet (HFD) for 20 weeks, alone or with a daily IP of 10 μmol/Kg fluorocitrate (FC) to investigate the enteric glia involvement in HFD-induced neuropathology. (B) Weekly body weight gain exhibited by standard diet (SD) and high-fat diet (HFD) mice during the 20 weeks of diet protocol. Data were analyzed by 2-way ANOVA and Dunnettpost-hoc. Results are expressed as average weights than their SD counterparts. [file 12974_2021_2164_MOESM1_ESM.tif]

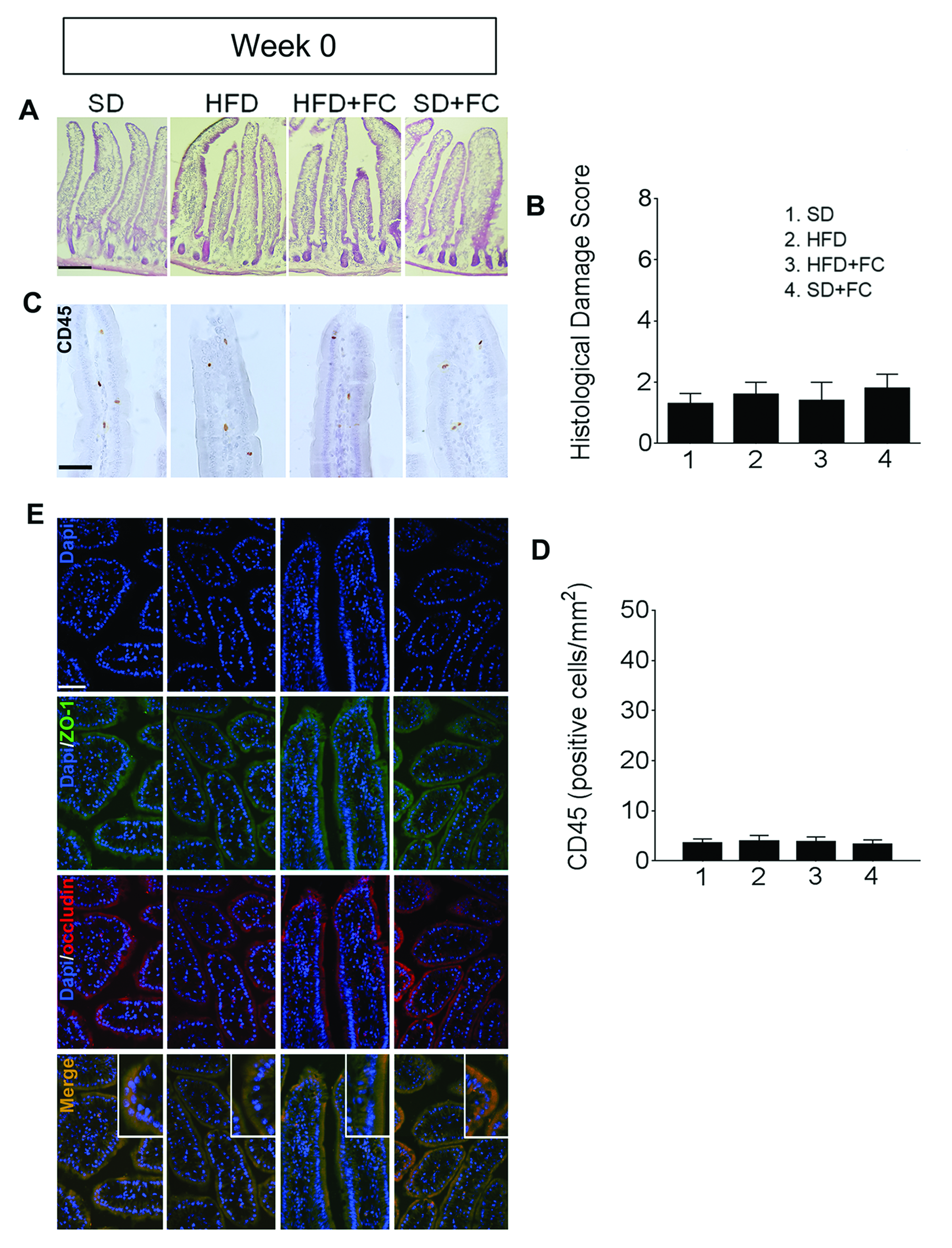

Supplement: Supplementary file 2 — Additional file 2 Representative images of duodenal cross-sections stained before starting the diet protocol. Representative images of duodenal cross-sections stained by (A) hematoxylin and eosin or immunolabeled for (C) CD45 (brown) or (E) Dapi (blue), ZO-1 (green), and occludin (red) before starting the diet protocol. (B) Relative total histological damage score, (D) average number of CD45+ cells/mm2. Data were analyzed by 2-way ANOVA or 1-way ANOVA and Dunnettpost-hoc. Results are expressed as cumulative histological damage score ± SEM or the average relative fluorescence units (RFU) ± SEM per area unit of n assessments. Scale bars = 10 and 30 μm. [file 12974_2021_2164_MOESM2_ESM.tif]

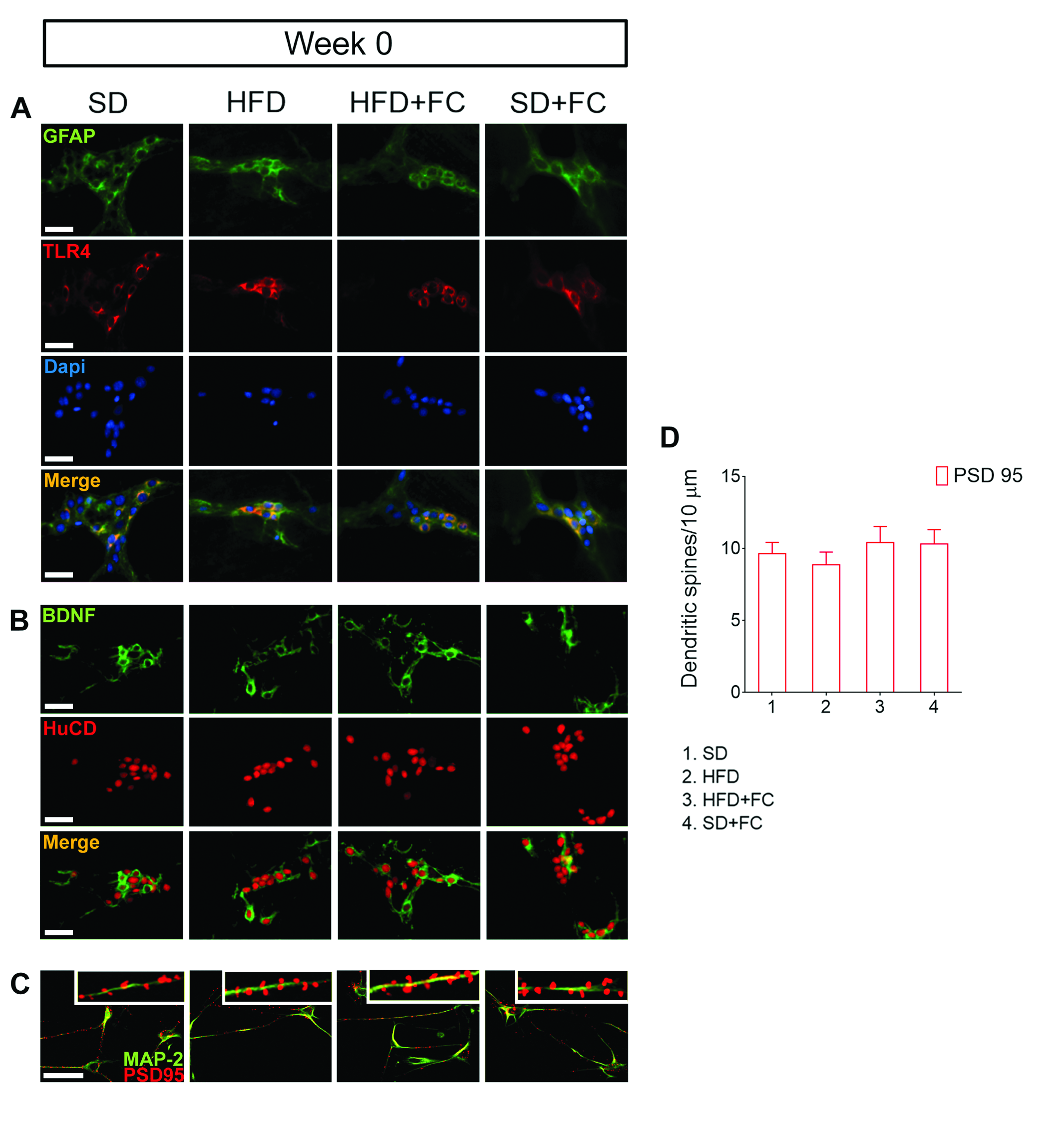

Supplement: Supplementary file 3 — Additional file 3 Representative images of duodenal myenteric plexus and cultured enteric neurons before starting the diet protocol. In the duodenal myenteric plexus, (A) GFAP (green), TLR4 (red), and Dapi (blue) immunoreactivity were quantified together with (B) BDNF (green) and HuCD (red) expression at week 0 (relative immunolabeling quantification shown in Fig. 2). The number of spines (PSD 95 immunoreactivity, red) was measured along neuronal dendrites (MAP-2 immunoreactivity, green) of cultured enteric neurons isolated from the duodenal myenteric plexuses before starting the diet protocol (C and D). Data were analyzed by 2-way ANOVA or 1-way ANOVA and Dunnettpost-hoc. Results are expressed as average relative fluorescence units (RFU) ± SEM per area unit or average number of dendritic spines/10 μm of n assessments. Scale bars = 10 and 20 μm. [file 12974_2021_2164_MOESM3_ESM.tif]

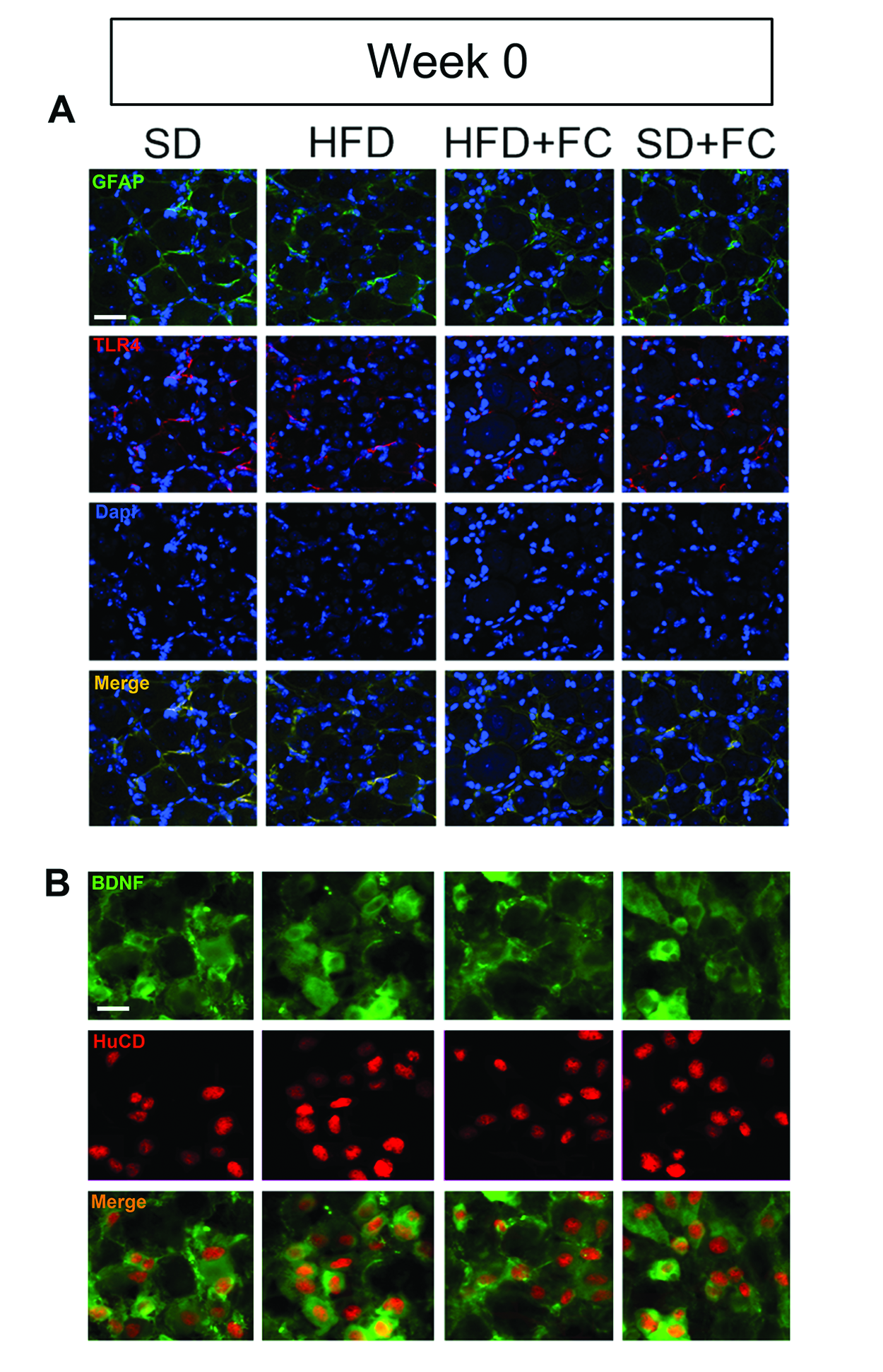

Supplement: Supplementary file 4 — Additional file 4 Immunofluorescence images of nodose ganglia isolated from each group before starting the diet protocol. (A) GFAP (green), TLR4 (red), and Dapi (blue) in the nodose ganglia and relative immunolabeling quantification shown in Fig. 3. (B) BDNF (green) and HuCD (red) expression in nodose ganglia with relative quantification is also shown. Data were analyzed by 2-way ANOVA and Dunnettpost-hoc. Results are expressed as average relative fluorescence units (RFU) ± SEM per area unit of n assessments. Scale bar = 20 μm. [file 12974_2021_2164_MOESM4_ESM.tif]

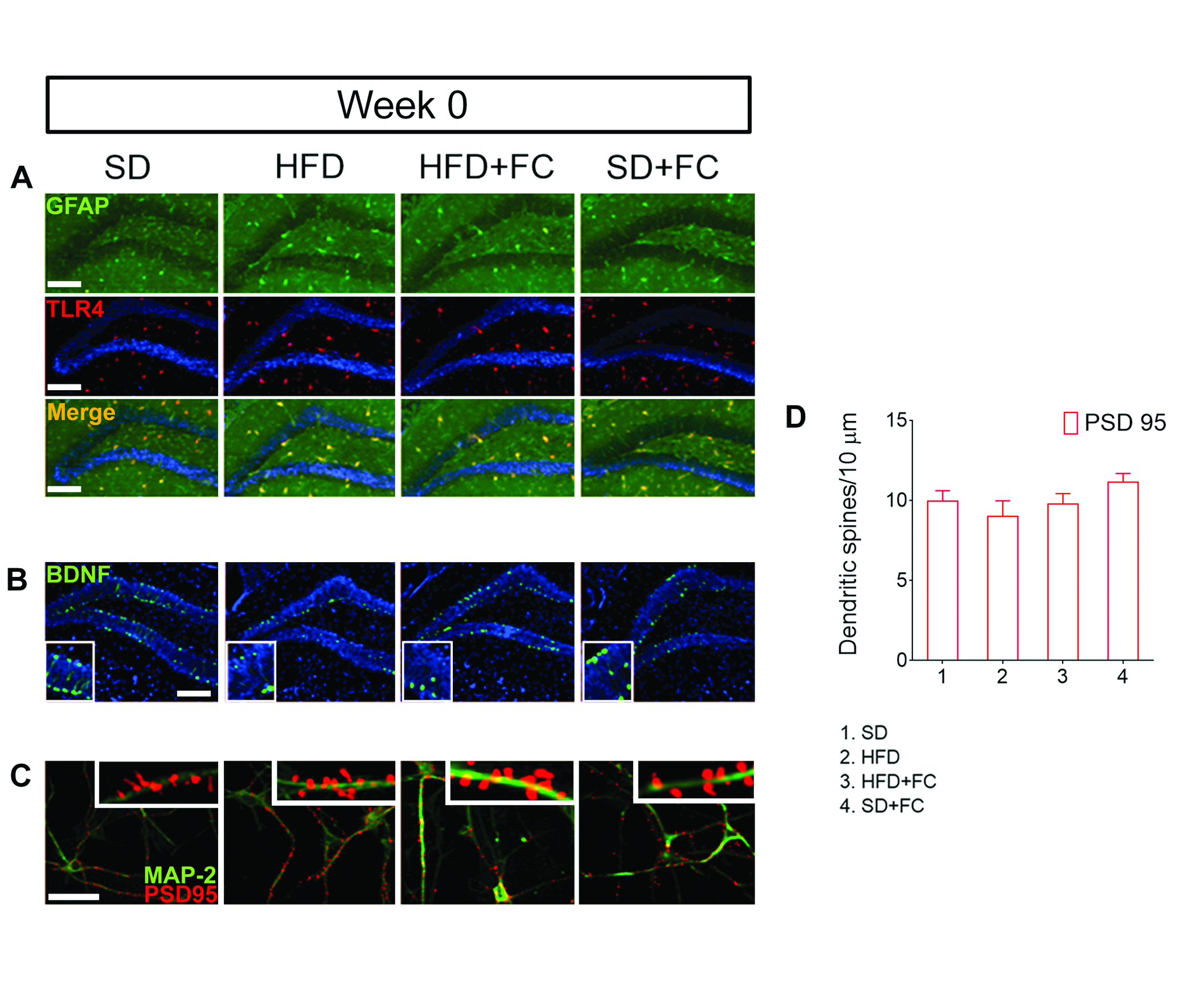

Supplement: Supplementary file 5 — Additional file 5 Representative images of hippocampal dentate gyrus and cultured neurons before starting the diet protocol. (A) Triple-label immunofluorescence for GFAP (green), TLR4 (red), and Dapi (blue) with relative fluorescence intensity quantification shown in Fig. 4. (B) BDNF protein expression (green) and immunoquantification in the dentate gyrus of the hippocampus. (C and D) Representative pictures show the neuronal spines (PSD 95 immunoreactivity, red) measured along hippocampal neurons’ dendrites (MAP-2 immunoreactivity, green) isolated before starting the diet protocol. Data were analyzed by 2-way ANOVA or 1-way ANOVA and Dunnettpost-hoc. Results are expressed as average relative fluorescence units (RFU) ± SEM per area unit or the average number of dendritic spines/10 μm of n assessments. Scale bars = 10 and 20 μm. [file 12974_2021_2164_MOESM5_ESM.tif]

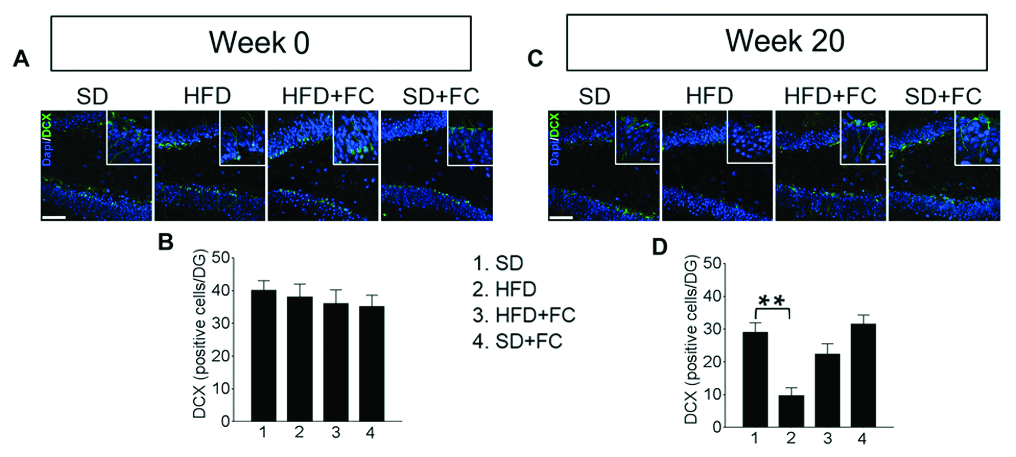

Supplement: Supplementary file 6 — Additional file 6 Immunofluorescence images showing the doublecortin X (DCX)-positive cells in the dentate gyrus of the hippocampus. Representative images for Dapi (blue) and DCX (green) with relative quantification of DCX-positive cells into the dentate gyrus area at (A-B) 0 and (C-D) 20 weeks. Data were analyzed by 1-way ANOVA and Dunnettpost-hoc. Results are expressed as average number of DCX-positive cells ± SEM in the dentate gyrus of n assessments. Scale bar = 100 μm. [file 12974_2021_2164_MOESM6_ESM.tif]
